# Therapy Combining Sorafenib and Natural Killer Cells for Hepatocellular Carcinoma: Insights from Magnetic Resonance Imaging and Histological Analyses

**DOI:** 10.3390/cancers17040699

**Published:** 2025-02-19

**Authors:** Zigeng Zhang, Guangbo Yu, Aydin Eresen, Qiaoming Hou, Sha Webster, Farideh Amirrad, Surya Nauli, Zhuoli Zhang

**Affiliations:** 1 Department of Radiological Sciences, University of California Irvine, Irvine, CA 92617, USA; aeresen@hs.uci.edu (A.E.); qiaominh@hs.uci.edu (Q.H.); 2 Department of Biomedical Engineering, University of California Irvine, Irvine, CA 92617, USA; guangboy@uci.edu; 3 Chao Family Comprehensive Cancer Center, University of California Irvine, Irvine, CA 92617, USA; 4 Department of Biomedical and Pharmaceutical Sciences, Harry and Diane Rinker Health Science Campus, Chapman University, Irvine, CA 92618, USA; shwebster@chapman.edu (S.W.); amirrad@chapman.edu (F.A.); nauli@chapman.edu (S.N.); 5 Department of Medicine, University of California Irvine, Irvine, CA 92617, USA; 6 Department of Pathology and Laboratory Medicine, University of California Irvine, Irvine, CA 92617, USA

**Keywords:** hepatocellular carcinoma (HCC), sorafenib, natural killer (NK) cells, combination therapy, tumor fibrosis, magnetic resonance imaging (MRI)

## Abstract

**Background/Objectives:** Hepatocellular carcinoma (HCC) remains a significant global health issue due to its high mortality rate and resistance to standard treatments. Sorafenib, the first-line systemic therapy for unresectable HCC, shows limited effectiveness due to resistance and severe side effects. Recent studies suggest that combining sorafenib with immunotherapy, particularly natural killer (NK) cells, may improve treatment outcomes. **Methods:** This study examined the effectiveness of sorafenib combined with NK cells pretreated with interleukin-12 (IL-12) and interleukin-18 (IL-18) in a rat HCC model. Tumor progression and treatment outcomes were assessed using MRI and histological analysis. **Results:** The results show that combination therapy significantly reduced tumor growth, increased tumor cell density, and inhibited angiogenesis and fibrosis in the tumor microenvironment. The sorafenib- and IL-12/IL-18-pretreated NK cell combination enhanced tumor inhibition by overcoming drug resistance and modulating the immune response. **Conclusions:** This study suggests that this combination therapy could be a promising strategy for treating HCC, offering both direct antitumor effects and modification of the tumor microenvironment for better clinical outcomes.

## Simple Summary

Hepatocellular carcinoma (HCC) is the sixth most common cancer worldwide and has a poor prognosis, with a five-year survival rate of 18%. Late-stage diagnosis HCC limits the curative options such as liver transplantation or resection, making systemic therapy the primary treatment. Sorafenib, an FDA-approved multikinase inhibitor, suppresses tumor proliferation and angiogenesis. However, its clinical efficacy is hindered by drug resistance and adverse effects, leading to suboptimal outcomes. Strategies combining sorafenib with other agents have been explored but with limited success. Immunotherapy, particularly natural killer (NK) cells, has emerged as a potential treatment due to their cytotoxic activity and immune-modulating effects. Studies indicate that preactivation with interleukin-12 (IL-12) and interleukin-18 (IL-18) enhances NK cell function. This study investigates the therapeutic potential of sorafenib combined with preactivated NK (PNK) cells in HCC.

## 1. Introduction

Hepatocellular carcinoma (HCC), the most common form of primary liver cancer, accounts for more than 90% of all primary liver tumors. It is closely associated with cirrhosis, with 80–90% of patients with cirrhosis eventually developing HCC [1]. Globally, HCC ranks as the sixth most common cancer and is the third leading cause of cancer-related death. Its prognosis is poor, with a five-year survival rate of just 18%, making it second only to pancreatic cancer in terms of lethality [2,3,4]. Due to its often asymptomatic and difficult-to-detect nature, HCC is frequently diagnosed in the advanced stages, with many patients already presenting with extrahepatic metastases. As a result, the opportunity for curative treatments such as liver transplantation or surgical resection is often missed for these patients. For this group of patients, systemic therapy has become the primary treatment option [5].

Sorafenib, a systemic treatment, was the first drug approved by the FDA for the targeted treatment of unresectable HCC and remains the first-line therapy. It operates by inhibiting the RAF/MEK/ERK pathway, directly impeding tumor cell proliferation as well as targeting vascular endothelial growth factor receptor (VEGFR) and platelet-derived growth factor receptor (PDGFR) to inhibit angiogenesis [6,7,8,9]. However, due to the high drug resistance of HCC and the adverse events associated with sorafenib, the median survival rate for patients who progress to the terminal stages is less than 10% [10,11,12,13]. Therefore, combination therapies are needed to overcome these limitations and improve treatment efficacy.

Researchers have investigated combining sorafenib with different drugs, including doxorubicin, selumetinib, interferon, capecitabine, and tegafur-uracil, but the results have been poor [14]. With advancements in immunology, attention has shifted to combining sorafenib with immunotherapy. Notably, some researchers have focused on natural killer (NK) cells, as these cells can directly kill tumor cells and participate in immune monitoring [15]. NK cells, integral to the body’s innate immune response, can identify and eliminate aberrant cells, including tumor cells, without prior sensitization. This inherent capability positions NK-based adoptive transfer immunotherapy as a promising avenue in HCC treatment [16]. Moreover, recent studies showcasing the augmented cytotoxicity of NK cells when combined with certain interleukins have provided additional avenues for exploration [17]. Hence, our experiment aimed to investigate the efficacy of interleukin-12 (IL-12)- and interleukin-18 (IL-18)-pretreated NK cells (PNK) in conjunction with sorafenib [18,19,20].

The rationale for selecting sorafenib and PNK stemmed from emerging research that suggests a synergistic relationship between sorafenib and NK cells. Sorafenib’s ability to weaken tumor cells could enhance the immunomodulatory effects, potentially increasing NK cell activity and strengthening the overall immune response against tumors [18,21]. To evaluate the therapeutic outcomes of sorafenib combined with NK cell immunotherapy in HCC, we utilized MRI as a non-invasive method, supplemented by histological analysis.

In our previous studies, we demonstrated that sorafenib, when combined with locally administered NK cells, effectively inhibited the growth of HCC. The invasiveness of intrahepatic NK cell delivery enhances the presence of NK cells in the tumor microenvironment but poses a challenge to the feasibility of multiple doses. To expand upon these findings and further refine this approach, we investigated the potential of sorafenib plus PNK cell therapy against HCC through a preclinical study focusing on a more stable hepatoma model, preferring a clinically applicable administration approach. This proposed approach aims to build on our prior results and assess whether a broader application of this combination could yield more consistent or enhanced therapeutic outcomes.

## 2. Materials and Methods

### 2.1. Experimental Procedure

**Figure 1:**
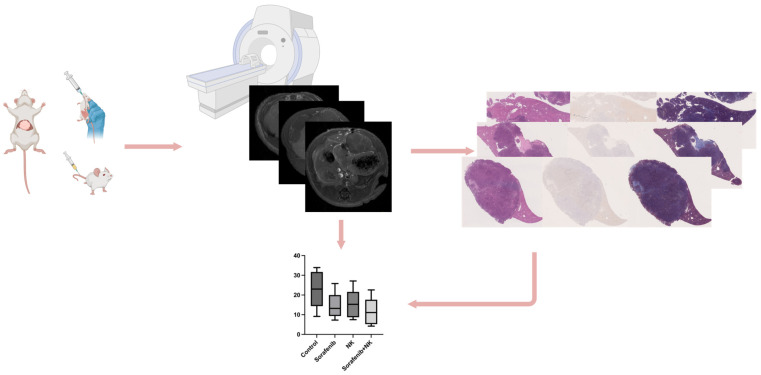
Experimental workflow. Tumors were implanted into rat livers on day 0. MRI scans were performed one week later, and treatment was initiated when the tumors reached approximately 5 mm in diameter. MRI scans were then performed weekly for three weeks to monitor tumor progression and treatment efficacy. After the scans, tumor samples were fixed and stained for analysis.

On day 1 of the experiment, McA-RH7777 cells were implanted orthotopically into the liver of Buffalo rats. MRI scans were performed one week later. When the maximum diameter of the tumor in the horizontal plane reached 5 mm, treatment was initiated immediately. MRI scans were then conducted weekly for three weeks to monitor tumor progression and treatment effects (as shown in Figure 1).

### 2.2. Cell Lines and Culturing

McA-RH7777 (McA), a rat hepatoma cell line (ATCC, CRL-1601, Manassas, VA, USA) [22], was cultured in Dulbecco’s modified Eagle’s medium (DMEM, ATCC, Manassas, VA, USA) supplemented with 10% FBS at 37 °C with 5% CO_2_. Before each tumor implantation, 2 tubes (about 0.5 mL) of McA cells were taken out from each flask. Then, 10 μL of cells from each tube was mixed with the same volume of Trypan blue at room temperature and then counted immediately to confirm a cell viability of more than 90%.

Rat NK cell line (generously provided by Dr. Thomas L. Olson, University of Virginia, Charlottesville, VA, USA) was cultured in 20 mL of RPMI medium per flask (Life Technologies, Waltham, MA, USA). The RPMI medium was supplemented with 25 mM 2-mercaptoethanol to maintain cell health and function. The cells were cultured at 37 °C in an environment with 5% CO₂. Before cell injection, the rat NK cell line was exposed to recombinant mouse IL-12 and IL-18 for 24 h. Following pretreatment, the cells were thoroughly washed with PBS to remove any remaining compounds and then transferred to a fresh medium. The same cell counting method used for McA cells was applied. The rat NK cells were deemed ready for injection if their viability exceeded 90% after pretreatment.

### 2.3. Animal Model

Orthotopic tumor implantation was performed in the livers of 24 rats, which were then randomly assigned to four groups: control, sorafenib, NK, and sorafenib plus NK, with six rats in each group.

A total of 1.5 × 10^6^ McA cells were suspended in 0.15 mL of a 1:1 Matrigel (Stemcell Technologies, Vancouver, BC, Canada) and PBS (Gibco, Grand Island, NY, USA) mixture. This cell suspension was then injected into the superficial layer of the liver of each rat. When the implantation was successful, a white bulge appeared at the injection site. Bloodstop (LifeScience Plus, Mountain View, CA, USA) hemostat was used to stop bleeding after injection. After confirming that there was no leakage and bleeding, the surgical site was closed layer-by-layer by using absorbable and non-absorbable sutures.

The treatment of the subjects began when the maximum diameter of the tumor was approximately 5 mm. In the sorafenib group and the sorafenib plus NK cell group, sorafenib mesylate was diluted in a 1:1 mixture of castor oil (Kolliphor^®^ EL, Sigma Aldrich, St. Louis, MO, USA) and 95% ethanol and administered orally using a ball-tipped gastric feeding needle. The daily dose was 10 mg/kg for seven consecutive days, once a day.

Rats in the NK cell immunotherapy group and the sorafenib plus NK cell therapy group received tail vein injections. Before injection, the rats were anesthetized with a mixture of air and oxygen (75/25% vol/vol, 2 L/min) with 1–2% isoflurane (VetOne, Boise, ID, USA), and the tail of the rat was warmed for 5–10 min using a warm pad or warm water to dilate the tail vein. After the placement of the needle, 0.1 mL of heparin was injected, followed by 10^7^ rat NK cells suspended in 0.3 mL of PBS and then 0.5 mL of PBS. Afterward, the needle was retracted, and pressure was applied to stop bleeding.

### 2.4. MRI

One week after tumor implantation, MRI scans were performed using a 3T Philips Achieva clinical MRI scanner (Philips Healthcare, Best, Netherlands) equipped with a commercially available wrist coil. Treatment was initiated when the tumor reached a maximum diameter of 5 mm on T2-weighted (T2w) MRI images. If the tumor did not reach 5 mm after the first scan, scans were performed daily until the tumor reached approximately 5 mm. After treatment, rats were monitored with MRI weekly until the third week after treatment to examine tumor growth and assess in vivo treatment response.

The imaging protocol involved T1-weighted (T1w) and T2w MRI sequences to monitor tumor growth and treatment efficacy. The MRI parameters used for image acquisition included an echo time (TE) of 63 ms, a voxel size of 0.6 mm × 0.6 mm, a slice thickness of 2 mm, and no inter-slice gap. The field of view (FOV) was optimized to range from 5 cm to 6 cm.

After MRI images were acquired, tumor tissue was delineated from T1w and T2w MRI data using ITK-SNAP (version 4.0, www.itksnap.org). Visualization and statistical analysis were performed using Python (v3.9, www.python.org) and Graph Prism (v.10.2, Boston, MA, USA).

### 2.5. Histology

After the final MRI scan, the researchers harvested the liver tissue for histological examination. We made sure the tissue slices were oriented with the MRI planes. Typically, one or two tissue sections, including the identified lesions and normal liver tissue, were extracted. These sections were fixed in a 10% formalin solution and alcohol of varying concentrations to preserve tissue morphology. The samples were processed at the Experimental Tissue Shared Resource Facility at the University of California, Irvine. Afterward, fixed tissue was embedded in paraffin for sectioning. Using a rotary microtome, the sections were cut to a thickness of 3 to 5 microns for histological analysis. The sections were mounted on glass slides and handled carefully to avoid damage or misalignment.

Hematoxylin & eosin (HE) staining was used to evaluate tumor burden. The tissue images were opened in QuPath (v0.5.1, qupath.github.io), and the grid function was enabled. A grid cell, representing a fixed area (unit area), was selected from the upper, lower, left, and right regions of the section. The average cell density per unit area was calculated by counting the cells within five grid cells. Each grid cell was considered a “unit area” for cell counting.

CD31 immunohistochemical staining was used to assess tumor angiogenesis. In QuPath, the numbers of CD31-positive and CD31-negative cells were counted. The ratio of CD31-positive to CD31-negative cells was calculated.

Fibrosis in the tumor was evaluated with Mason’s trichrome staining. In QuPath, the tumor area was outlined, and the fibrotic regions were identified. The ratio of fibrotic tissue to total tumor area was calculated. These staining techniques helped with analyzing the structural and cellular changes in HCC and its response to treatment. All images were analyzed using Python for statistical calculations and further visualization.

### 2.6. Statistical Analysis

All statistical analyses were performed using Python and GraphPad Prism. Data are presented as mean ± standard deviation (SD). The Shapiro–Wilk test was used to check normality. If the data followed a normal distribution, one-way ANOVA was applied, followed by Tukey’s post hoc test for multiple comparisons. If the data were not normally distributed, the Kruskal–Wallis test was used, followed by Dunn’s multiple comparison test. A *p*-value < 0.05 was considered statistically significant. Box plots were generated using the Matplotlib and Seaborn libraries in Python.

## 3. Results

### 3.1. MRI Results

#### Initial Tumor Assessment and Treatment Monitoring

Initial Tumor Assessment and Treatment Monitoring

**Figure 2:**
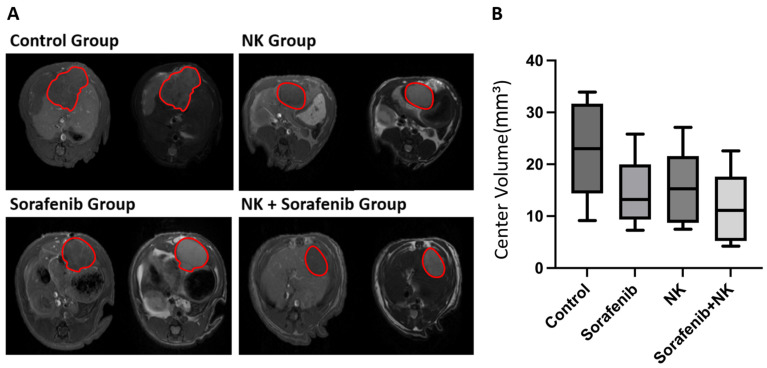
Tumor localization and growth monitoring. (**A**) T1-weighted (T1w) and T2-weighted (T2w) MR images used for tumor localization. The T1w image highlights the anatomical structures, while the T2w image provides enhanced contrast between the tumor and surrounding tissues. The tumor region is indicated by the red circled area. (**B**) According to the tumor contour on the T2w image in (**A**), the tumor on all slices was outlined. The slice with the largest cross-section as well as one slice before and after were selected from all tumors in the first and last weeks to calculate the central volume and compare the central volumes of the two weeks. The box plot illustrates the central volume ratio of the tumors in the four experimental groups (control group, NK group, sorafenib group, and sorafenib plus NK group).

The T1w and T2w MRI data were acquired when the maximum diameter of the tumor reached approximately 5 mm, which served as the baseline for this experiment, to assess tumor growth and the effectiveness of the treatment. As shown in Figure 2A, the difference in signal intensity between the normal liver tissue and tumor tissue allowed accurate localization of the tumor. Tumor tissue typically exhibits low signal intensity on T1w MRI and high signal intensity on T2w MRI, which helps distinguish it from normal liver tissue.

Following the acquisition of baseline images, treatment was initiated immediately. Post-treatment, MRI scans were performed every 7 days for 2 weeks. In the final week of the experiment, all baseline and last imaging layers were masked to isolate the tumor. The mask with the largest area and the masks of the two preceding and following slices were selected. The volumes of these three masks were summed and averaged to obtain the central volume of the tumor for both the baseline and last scans. The ratio of the central volume of the tumor in the final scan to the baseline was calculated for each rat (see Figure 2B).

As illustrated in Figure 2B, although no significant difference was found in the tumor volume ratio between the single treatment groups and the combined treatment group, a considerable difference was observed. The combined treatment group showed a greater reduction in tumor volume than the single treatment groups. This suggests that combining therapies had an added or enhanced effect. Although the difference was not statistically significant, it could still be biologically important.

### 3.2. Histological Analysis

#### 3.2.1. Hematoxylin and Eosin Staining Analysis

**Figure 3:**
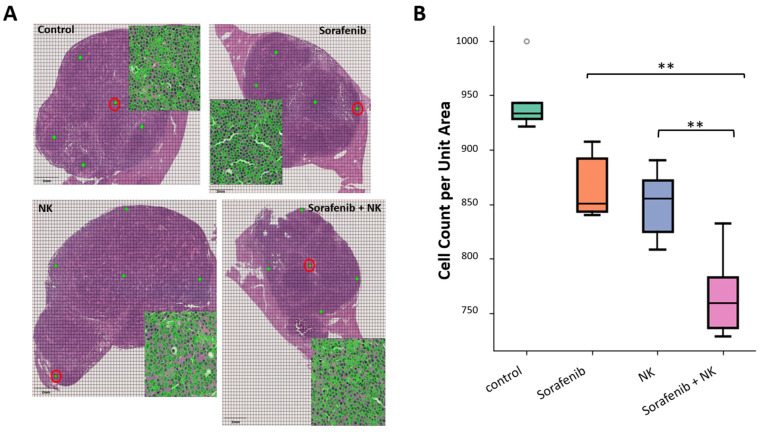
HE staining and cell count per unit area. (**A**) A grid (representing a unit area) was randomly selected from the top, bottom, left, or right side of the tumor for cell counting, and the number of cells in each grid was calculated. The image also includes a magnified view, and the magnified area is highlighted with a red circle. The enlarged unit area is marked with a red circle, and the green dots show five-unit areas randomly chosen from the top, bottom, left, and right of the HE-stained slice. (**B**) Box plots show the number of cells per unit area for each experimental group. (**: *p* < 0.01).

To assess the effects of the treatments on tumor cell density and function, we performed HE staining of tumor sections. Figure 3A shows a selection of five-unit areas within each slide for cell counting, from which the average cell count was calculated. Tumor cell density is an important indicator of tumor proliferation and burden. In Figure 3B, we present the box plots for each treatment group. Significant differences between the sorafenib and sorafenib plus NK cell groups, as well as between the NK cell and sorafenib plus NK cell groups, are shown. These results suggest that sorafenib plus NK cell treatment could effectively reduce tumor cell density compared to sorafenib alone and NK cell alone, indicating its role in inhibiting tumor cell proliferation and improving tumor function.

#### 3.2.2. CD31 Staining Analysis

**Figure 4:**
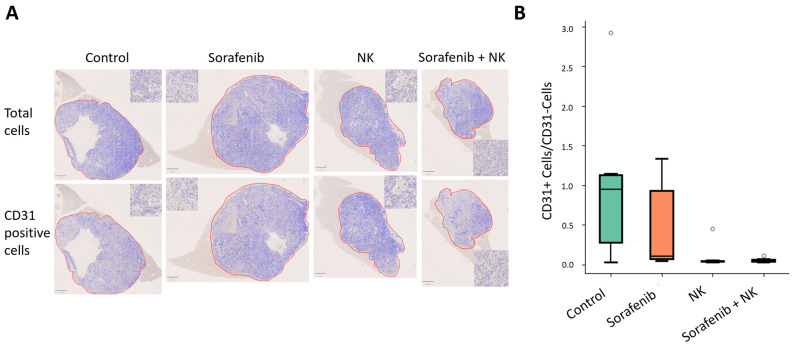
CD31 staining and cell quantification in tumor tissues. (**A**) Two images of CD31 staining for each group. The first image shows the total cell count, and the second image shows the number of CD31-positive cells. Cell counting was performed using QuPath, first outlining the tumor area and then using QuPath’s built-in counting function to count all cells and CD31-positive cells. The red outlined area is the tumor tissue. (**B**) Box plot of the ratio of CD31+ cell number to CD31- cell number in the Control group, Sorafenib group, NK group, and Sorafenib + NK group.

To evaluate the effect of the treatments on tumor vasculature, we performed CD31 staining of the tumor sections. Figure 4A shows CD31-positive cells and the total cells across groups. As shown in Figure 4B, we calculated the ratio of CD31-positive to CD31-negative cells in each group. Statistical analysis showed significant differences between the sorafenib and sorafenib plus NK cell treatment groups. These results suggest that sorafenib plus NK treatment more effectively regulates tumor vasculature compared to sorafenib alone. The inhibition of tumor vasculature, as seen in the combined treatment group, may limit the nutrient and oxygen supply to the tumor, reducing tumor growth and enhancing treatment efficacy by disrupting its ability to sustain itself.

#### 3.2.3. Masson’s Trichrome Staining Analysis

**Figure 5:**
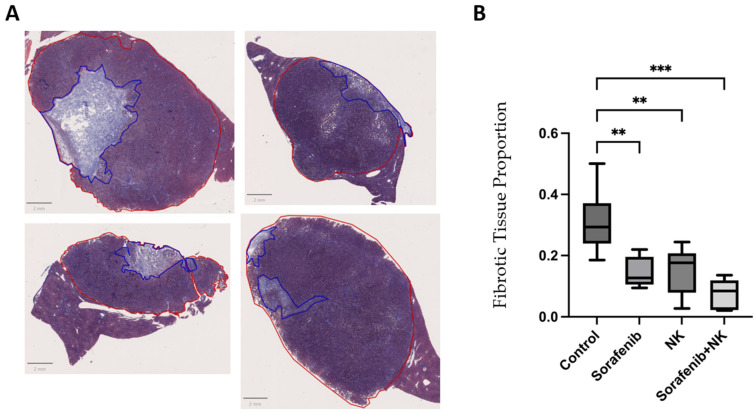
Trichrome staining and fibrotic area ratio. (**A**) First, the tumor area was outlined in QuPath, and then the fibrotic area was outlined (the red outline represents the tumor, and the blue outline represents the fibrotic area). The ratio of the fibrotic area to the total tumor area was then calculated. (**B**) The box plot shows the fibrotic area ratio for each experimental group. (**: *p* < 0.01; ***: *p* < 0.001).

To assess the effects of combined treatment on the tumor microenvironment, we performed Masson’s trichrome staining of the tumor tissues. This staining highlighted the fibrotic tissue, as shown in Figure 5A, where collagen fibers were stained blue with aniline blue. Fibrous tissue was clearly differentiated from cell nuclei, which were stained dark blue, and cytoplasm, which was stained orange-red. For quantitative analysis, we used QuPath (v.0.5.1) to measure the areas of tumor tissue and fibrotic tissue. One-way ANOVA revealed significant differences between the control and experimental groups (Figure 5B). Significant differences were found when comparing the single treatment groups with the combined sorafenib and NK cell treatment group. These results indicate that the combined treatment more effectively regulates the tumor microenvironment by reducing fibrosis and preventing tumor cell proliferation compared to single-agent treatment.

## 4. Discussion

In recent years, NK cells have gained significant attention in cancer research due to their intrinsic ability to target and regulate tumor cells. As key components of the innate immune system, NK cells act as the first line of defense against tumors. Interestingly, the percentage of NK cells in the liver is at least five times higher than in the spleen or peripheral blood, suggesting that NK cells may play a crucial role in the liver’s immune function. However, the therapeutic effect of NK cells has not met expectations in recent years [23].

Recent studies have found that activated NK cells have a better tumor suppression function than ordinary NK cells. The study of Zhuang et. al. demonstrated that NK cells activated by a cytokine mix of IL-12, IL-15, and IL-18 showed enhanced cytotoxicity against HCC cell lines in vitro [24]. In the study, flow cytometry was utilized to examine the changes in NK cell surface receptors, both activating and inhibitory, before and after activation. Moreover, the cytotoxicity of the activated NK cells against different human HCC cell lines was evaluated in vitro. Finally, the activated NK cells were injected into an animal model with spontaneous HCC to see if these NK cells would migrate to the liver and fight the tumor. While IL-12, IL-15, and IL-18 enhanced NK cell function, further optimization is required for maximum therapeutic efficacy.

In contrast, our study focused on the responses of tumor cells and their microenvironment after combination therapy. We pretreated rat NK cells (RNK-16) with IL-12 and IL-18 and combined the activated NK cells with sorafenib, a first-line drug for the treatment of unresectable HCC. Our results showed that this combined therapy not only significantly inhibited tumor cell proliferation but also induced profound changes in the tumor microenvironment. Compared with activated NK cell treatment alone, this strategy enhanced tumor suppression by overcoming sorafenib resistance and improving NK cell function.

The histopathological analysis further confirmed these findings. HE staining showed a reduction in tumor burden, while CD31 immunohistochemistry showed reduced tumor vascularization, indicating impaired angiogenesis. In addition, trichrome staining highlighted the reduction in fibrosis in the tumor microenvironment. These results suggest that the combination of IL-12/IL-18-pretreated NK cells with sorafenib can not only directly inhibit tumor cells but also modulate the tumor microenvironment, ultimately leading to more favorable treatment outcomes.

In addition to improving treatment options, we also explored non-invasive tumor monitoring methods. In our study, we utilized T1w and T2w MRI for tumor growth monitoring. MRI was chosen as the primary imaging modality due to its superior soft tissue contrast and ability to provide detailed anatomical information without the use of ionizing radiation [25]. This makes MRI particularly suitable for long-term monitoring, as it eliminates the risks associated with repeated exposure to ionizing radiation, such as mutations, cell death, and potential cancer development, as highlighted by Semelka et. al. in 2007 [26]. Furthermore, MRI’s use of strong magnetic fields and radiofrequency pulses allows for excellent differentiation between soft tissue structures, which is critical for accurate tumor assessment. Given these advantages, MRI remains the preferred imaging modality for patients requiring prolonged and repeated tumor monitoring.

## 5. Conclusions and Future Perspectives

In this study, we showed that combining IL-12/IL-18-pretreated NK cells with sorafenib improved the inhibition of tumor growth, function, and fibrosis in a rat model of HCC compared to each treatment alone. Our results highlight the potential of this combination of therapy to overcome the limitations of sorafenib, especially in drug-resistant tumors, by boosting NK cell cytotoxicity. Additionally, using MRI as a non-invasive tool to monitor tumor progression and treatment response provides a more reliable alternative to CT imaging.

In the future, more research is needed to optimize the clinical use of this combined therapy. Studies should explore different administration routes, the timing of NK cell activation, and the possible synergies between sorafenib and other immune-modulatory agents. Expanding research into other tumor models and eventually human clinical trials will be important to confirm these results. Moreover, progress in imaging methods, including MRI, could improve tumor monitoring and enable real-time adjustments to treatment. In conclusion, combining targeted therapies with advanced immunotherapy offers a promising approach to improve outcomes for patients with otherwise untreatable HCC.

## Data Availability

The data that supports the findings of this study are available from the authors upon reasonable request.
